# Tissue oxygenation dynamics during transition from seizure to spreading depolarization in rat brain

**DOI:** 10.1002/epi.70207

**Published:** 2026-03-20

**Authors:** Jiayang Liu, Bruce J. Gluckman

**Affiliations:** ^1^ Neural Engineering Center Pennsylvania State University University Park Pennsylvania USA; ^2^ Engineering Science and Mechanics Department Pennsylvania State University University Park Pennsylvania USA; ^3^ Neurosurgery Department Pennsylvania State College of Medicine Hershey Pennsylvania USA; ^4^ Biomedical Engineering Department Pennsylvania State University University Park Pennsylvania USA

**Keywords:** epilepsy, extracellular space, pulse voltammetry, spreading depolarization

## Abstract

**Objective:**

Spreading depolarization (SD) is a phenomenon underlying various neurological conditions, including epilepsy. Researchers have suspected that local tissue oxygenation breakdown induces spontaneous SD. In this study, we investigated the relationship between spontaneous epileptic seizures and SD, with a focus on the role of local tissue oxygenation during the transition from seizure to seizure‐associated SD.

**Methods:**

We applied a long pulse voltametric method to characterize local tissue oxygenation and extracellular space (ECS) volume change in the hippocampus (HC) of freely moving epileptic rats (six males and three females). Recordings were performed during the normal state of vigilance, spontaneous seizures, seizure‐associated SD events, and their transitions.

**Results:**

No significant breakdown in local tissue oxygenation of HC was detected before the SD onset during the seizure to SD transition. In contrast, decreased ECS volume in the HC was observed before SD onset during this transition.

**Significance:**

Using a novel electrochemical approach in freely behaving rats with intact cerebral autoregulation, we demonstrate that there is no significant breakdown of local tissue oxygenation during seizure to SD transition. However, the ECS begins to shrink during seizure, before SD onset, suggesting that ECS shrinkage may play a leading role in this transition. These findings refine our understanding of the mechanisms driving seizure‐associated SD and suggest that ECS may represent a potential therapeutic target in epilepsy and SD‐associated neurological disorders.

**Key points**

- Direct in vivo recordings using a long‐pulse voltametric electrochemical method in freely moving rats characterized local tissue oxygenation and ECS volume dynamics in normal states of vigilance, spontaneous seizures, seizure‐associated SD events, and their transitions.
- No significant breakdown in local tissue oxygenation was detected before SD onset during the seizure to SD transition, suggesting that overt tissue hypoxia is not the primary trigger for SD in this context.
- A decrease in ECS volume was observed before SD onset during the seizure to SD transition, suggesting that ECS shrinkage may play a potential leading role in SD initiation following seizures.
- ECS is the primary pathway for oxygen diffusion within brain tissue.
- These findings refine the mechanistic understanding of seizure‐associated SD and highlight ECS homeostasis as a potential therapeutic target for preventing SD and its pathological consequences in epilepsy.

## 1 INTRODUCTION

Although the human brain occupies only ~2% of the body's mass, it consumes ~20% of the body's oxygen for resting metabolism.^1^ Na^+^‐K^+^‐adenosine triphosphatase (ATPase) and other ATP‐dependent pumps consume a significant portion of that 20% to maintain the homeostasis of ion gradients across cell membranes. Arterial blood delivers oxygen, which diffuses from red blood cells through plasma, capillary walls, and the extracellular space (ECS) to surrounding cells. The ECS is the narrow microenvironment that surrounds every cell of the central nervous system and occupies approximately 20% of brain tissue.^2^ It is characterized by the volume fraction *α* and the tortuosity *λ*.^3^

Continuous and adequate oxygen supply, along with tight metabolic regulation, is essential for maintaining neuronal resting metabolism and signal processing. Spreading depolarization (SD) disrupts this homeostatic balance.^4^ SD is characterized by profound extracellular ionic shifts,^5^ sustained neuronal depolarization leading to depolarization block,^6^ and loss of electrical activity,^7^ as well as cell swelling and distortion of dendritic spines.^8^ SD is recorded as a high‐amplitude negative direct current shift of the slow potential.^9^ During SD, neurons cannot fire because of sustained depolarization blocks, causing an almost complete electrical silence named spreading depression in the cortex.^7^ In other words, spreading depression is a consequence of SD resulting from a depolarization block of neuronal activity.^4^

SD has been reported as the phenomenon underlying migraine aura^10^, ^11^, ^12^ and appears sufficient to activate trigeminal nociception and sustain it for durations consistent with the migraine attack.^13^ SD is also closely associated with subarachnoid hemorrhage, intracranial hemorrhage,^10^, ^14^, ^15^ stroke, traumatic brain injury,^16^, ^17^ epilepsy and epileptogenesis,^15^, ^18^ and sudden unexpected death in epilepsy (SUDEP).^19^

SD can be induced directly by neuronal depolarization via sodium or calcium channel activation (e.g., glutamate or potassium application) or indirectly through reduced Na^+^/K^+^‐ATPase activity during ischemia, hypoxia, or hypoglycemia. Electrical or mechanical stimulation can also trigger SD.^20^ Techniques such as gradient‐echo magnetic resonance imaging (MRI), diffusion‐weighted MRI, and optical intrinsic signal (OIS) imaging have been implemented to study SD‐associated effects like cell swelling, cerebral blood volume/flow (CBV/CBF) change, and hemoglobin concentration fluctuation. Potassium chloride‐evoked SD in rats produced an increased magnetic resonance signal due to venous oxygenation increase.^21^ SD‐induced cell swelling reflected in tissue water apparent diffusion coefficients decreases and negatively correlates with blood oxygenation level‐dependent signal.^22^, ^23^ The ECS volume shrinkage due to SD‐induced cell swelling has also been demonstrated.^24^ OIS detects reflectance changes in light scattering caused by CBV/CBF, hemoglobin, and cytochrome redox fluctuations during SD under normal and pathological conditions.^25^, ^26^

SD consumes a large amount of oxygen, dramatically interferes with brain tissue oxygenation level, and represents a high‐energy challenge for brain tissue.^27^ The tissue oxygenation dynamics have been studied in different SD models,^8^, ^28^, ^29^, ^30^, ^31^ and recent two‐photon ATP imaging studies reveal transient neuronal ATP depletion during SD, highlighting its risk under ischemic conditions.^32^

A key limitation of previous studies is their reliance on induced SD models under nonphysiological conditions (e.g., ischemia or hypoxia), which limits evaluation of SD initiation and propagation under intact neurovascular regulation. Additionally, there has been inferred causation from correlation, for instance, interpreting hypoxia‐induced SD as typical seizure physiology, a potential interpretative error highlighted in Bernard.^33^ Modeling work emphasizes the importance of tissue oxygenation in seizure–SD interactions,^34^ and slice experiments have examined related oxygen dynamics.^35^ However, these works lack physiological autoregulation, intact metabolism, and neurovascular coupling regulation.

The ECS is increasingly recognized as a dynamic, actively regulated structure that modulates molecular diffusion and neuronal activity, positioning ECS modulation as a potential therapeutic target in neurological disorders.^36^

In a previous study, we used a tetanus toxin (TeTX) model of temporal lobe epilepsy, in which presynaptic inhibitory neurotransmitter release is disrupted, leading to spontaneous seizures and seizure‐associated SD.^37^ This model enables investigation of SD initiation and seizure–SD interactions under intact autoregulation. We demonstrated continuous, long‐term recordings of electrophysiology and electrochemistry during different state transitions, including spontaneous seizure to SD transitions.^38^ Here, we investigate the mechanisms underlying these transitions with intact blood flow autoregulation and metabolic control, with particular emphasis on the initiation of SD. In the TeTX model, when an animal enters a spontaneous seizure from a normal state of vigilance (SOV), that is, rapid eye movement (REM) sleep, non‐REM (NREM) sleep, or wake state, one of two trajectories follows: seizure termination or progression into SD. The mechanisms determining this divergence remain unclear. We examine tissue oxygenation across normal SOV, periseizure, and peri‐SD transitions using constant potential amperometry (CPA) and a new long pulse voltammetry (LPV). By applying the LPV, we characterize the local tissue oxygenation. We also extract and characterize the effective diffusion coefficient. Additionally, this approach enables an indirect characterization of ECS volume fraction changes during these transitions.

## 2 MATERIALS AND METHODS

### 2.1 One‐dimensional model of oxygen diffusion and consumption in brain tissue

**FIGURE 1:**
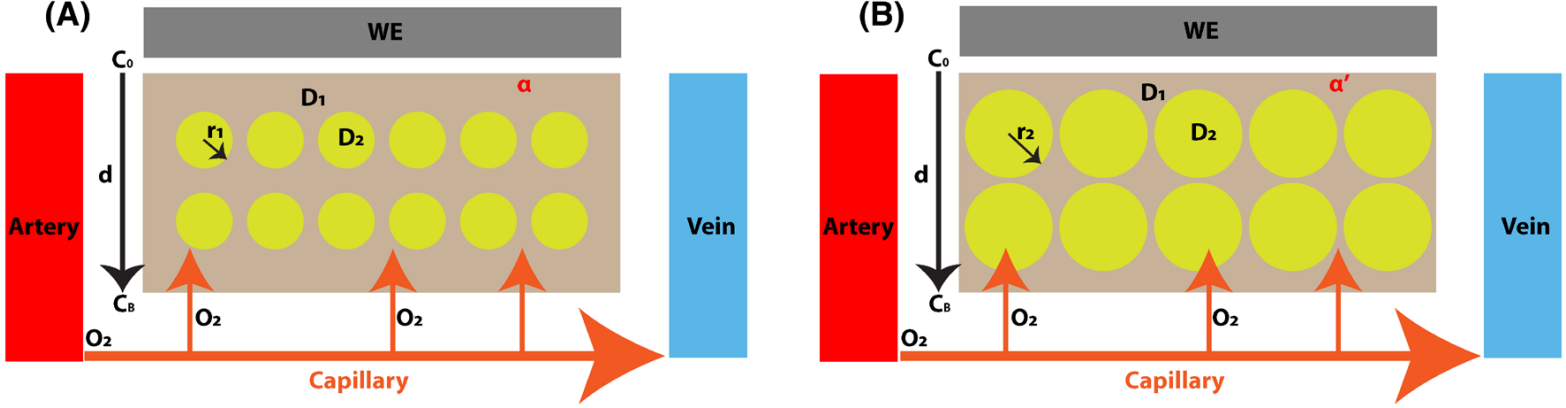
One‐dimensional model of oxygen diffusion in brain tissue. Oxygen molecules (O_2_) carried by red blood cells enter capillaries from the artery (red) to the vein (blue), are released from hemoglobin, and continuously diffuse into the interstitial fluid. (A) Normal state of vigilance. (B) Pathological state. *C*_0_, local oxygen concentration at the WE surface; *C*_B_, bulk oxygen concentration near the capillary; *d*, Nernst diffusion thickness; *D*_1_ */D*_2_, oxygen effective diffusion coefficient of ECS/cell; ECS, extracellular space; *r*_1_/*r*_2_, cell (yellow) radius; WE: platinum (Pt) working electrode; *α/αʹ*, ECS volume fraction.

Oxygen delivery to brain tissue depends on cardiorespiratory function and hemoglobin binding (~98% of total oxygen).^39^ Previous studies on diffusion models^40^, ^41^, ^42^ assume a uniform oxygen consumption rate, a constant effective diffusion coefficient (*D*_eff_), and even‐spaced capillaries. However, these assumptions break down during pathological states such as seizures or SDs, when (1) bulk oxygen concentration (*C*_B_) fluctuates with CBF/CBV and hemoglobin oxygenation and (2) *D*_eff_ is altered by changes in ECS volume fraction (*α*) and tortuosity (*λ*). To account for these dynamics, we implemented a one‐dimensional (1D) diffusion model of brain tissue oxygenation as shown in Figure 1A. Arteries deliver oxygen bound to hemoglobin within red blood cells. As blood flows into capillaries, oxygen is released from hemoglobin and diffuses into the interstitial fluid, and then from the bulk tissue (near capillaries) to the oxygen‐sensing site at the electrochemical working electrode (WE) tip. Because the WE tip surface area is much larger than individual cells, axial oxygen diffusion can be neglected. We assume a uniform oxygen consumption rate across tissue. The average distance between the WE surface (*x =* 0) and the nearest capillary is 25 μm on average in vivo, and typically 100 μm from the bath when recording in vitro.^34^ This distance (*d*) is assumed to be constant across different states, which include REM sleep, NREM sleep, wake, seizure, and SD event. Cell centers are assumed fixed. Cell radius (*r*) changes lead to *α* changes with *λ* assumed constant. Consequently, the *D*_eff_ is modeled as dependent on *α*.

#### 2.1.1 Equations when we consider oxygen reduction only at electrode‐to‐tissue interface

*Current from Fick's law (mass transport)*

The oxygen reduction faradic current at the interface (or diffusion current) is governed by the diffusion and for 1D model we have:

(1) $$J_{\text{diff}}=-\mathrm{nF}D_{\mathrm{eff}}{\left.\frac{\mathrm{d}C}{\mathrm{d}x}\right|}_{x=0}$$

where *n* is the number of electrons transferred and *F* is the Faraday constant.

### 2.2 Voltametric methods solutions

We applied CPA and LPV to the model. In CPA, a constant bias potential (−.65 V), that is, a large overpotential, is applied on WE with respect to the reference electrode and the resulting current is diffusion‐dependent, as described in Equation (1):

(2) $$i_{\mathrm{CPA}}={\mathrm{nFD}}_{\mathrm{eff}}\frac{C_{B}}{d}+i_{n}={\mathrm{AD}}_{\mathrm{eff}}C_{B}+i_{n}$$

where *i*_*n*_ represents nonfaradic current and other measurement noise, *A* is a geometric factor.

Shown in Equation (2), CPA yields a mixed signal of *C*_B_ and *D*_eff_. Studies using CPA assume a constant *D*_eff_ or cannot extract it from the mixed signal.^43^ Extracting the *D*_eff_ and characterizing its dynamics, especially during pathological states (e.g., seizures or SD events), have not been studied. To fill this gap, we applied the LPV.

**FIGURE 2:**
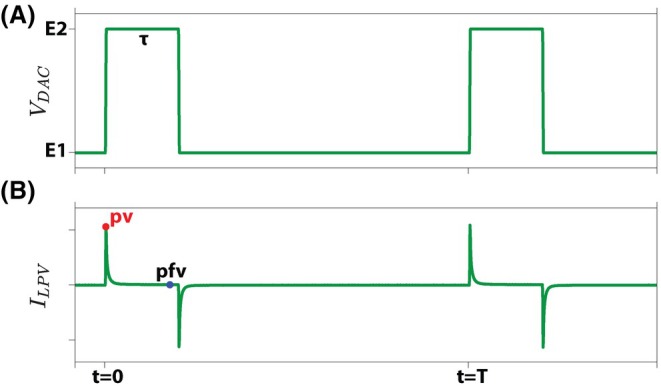
The long pulse voltammetry (LPV) waveform and current response example. (A) Bias potential waveform V from digital to analog converter (DAC). E1, potential in equilibrium region, E2, potential in mass‐transfer‐limited region, τ, pulse duration. (B) Response current I. pfv, LPV current response's peak flat value; pv: LPV current response's peak value; t, time, and T, period.

The LPV waveform is composed of periodic pulse potentials. A one‐period potential waveform and its current response are shown in Figure 2. Potential *E*_1_ lies in the region where an equilibrium state is held, and *E*_2_ lies in the “mass‐transfer‐limited” region where rapid oxygen reduction drives the local oxygen concentration at the WE surface nearly to zero. As the bias potential changes from *E*_1_ to *E*_2_, a large overpotential is applied, and the initial current response peak value (pv) is dominantly dependent on the initial oxygen concentration, which is *C*_B_, so:

(3) $$J_{\mathrm{pv}}=BC_{B}+i_{\mathrm{npv}}$$

where *i*_npv_ represents nonfaradic current and other measurement noise and *B* is a geometric factor.

After the initial current peak, current flows continue maintaining the fully oxygen reduced condition at potential *E*_2_, which becomes the CPA situation, and we get the current peak flat value (pfv):

(4) $$J_{\mathrm{pfv}}={\mathrm{AD}}_{\mathrm{eff}}C_{B}+i_{n}$$

To extract *D*_eff_, we assume:

1. Within each state (e.g., seizure or SD), $D_{\mathrm{eff}}=\bar{D}+\Delta D$, $C_{B}=\bar{C_{B}}+\Delta C_{B}$ and Δ*C*_B_ and Δ*D*_eff_ are dependent on its state and independent of each other.
2. *i*_*n*_ and *i*_npv_ are dependent on its state and independent of each other.

Based on Equations (3) and (4), we can get a ratio value (*r*) using the covariance of *J*_pv_ and *J*_pfv_ and the variance of *J*_pv_.

(5) $$\;r\propto \frac{\mathrm{cov}\left(J_{\mathrm{pfv}},J_{\mathrm{pv}}\right)}{\mathrm{var}\left(J_{\mathrm{pv}}\right)}=\frac{A}{B}D_{\mathrm{eff}}$$

From Equation (5), we can use *r* to characterize *D*_eff_ changes. For the same animal, *r* is state‐dependent. From one state to another (e.g., NREM to REM, or seizure to SD), we can extract *D*_eff_ by doing ensemble averages of the ratios with respect to transition times. Alternatively, for peri‐transition time ensembles, we take the variance and covariance at particular offset times with respect to the transition time directly across the events ensemble. The latter method has the advantage that the sample sizes are larger and the variances in concentration are, in practice, larger than the local noise terms. In the Results section, we report changes in CPA current (*I*_CPA_), LPV peak value (*I*_pv_), LPV peak flat value (*I*_pfv_), and *r*‐extracted *D* (or *D*_eff_) to quantify and illustrate our findings.

### 2.3 Relationship between ECS volume fraction and effective oxygen diffusion coefficient

Previous studies have demonstrated that cell membranes act as barriers to macromolecules but are partially permeable to small molecules such as oxygen.^44^, ^45^, ^46^, ^47^, ^48^, ^49^, ^50^ These studies have examined oxygen transport across cell membranes from the ECS into cells and the resulting concentration gradients.^43^ In brain tissue, oxygen diffuses through two parallel pathways: the ECS, with diffusion coefficient *D*_1_, and intracellularly, with diffusion coefficient *D*_2_ (Figure 1). Their relative contributions to the *D*_eff_ depend on cell morphology across brain states. During state transitions, cell morphology is modeled as changes in cell radius with a fixed cellular center (Figure 1B). As these changes are mainly driven by water flux, *D*_1_ and *D*_2_ are assumed constant, and with uniform oxygen solubility, *D*_eff_ can be expressed as:

(6) $$D_{\mathrm{eff}}=\alpha D_{1}+\left(1-\alpha \right)D_{2}$$

where *α* occupies ∼20% of the total volume.^2^ From Equation (6), changes in *D*_eff_ can be used to indirectly characterize alterations in ECS volume fraction *α*.

### 2.4 Animal surgery

All experimental protocols were approved by the institutional animal care and use committee. We used Long‐Evans rats (six males and three females) from Charles River Laboratories or Envigo, weighing 250–500 g.

Electrode implantation details, oxygen‐sensing electrode calibration, and data analysis and statistics are shown in Appendix S1.

## 3 RESULTS

**FIGURE 3:**
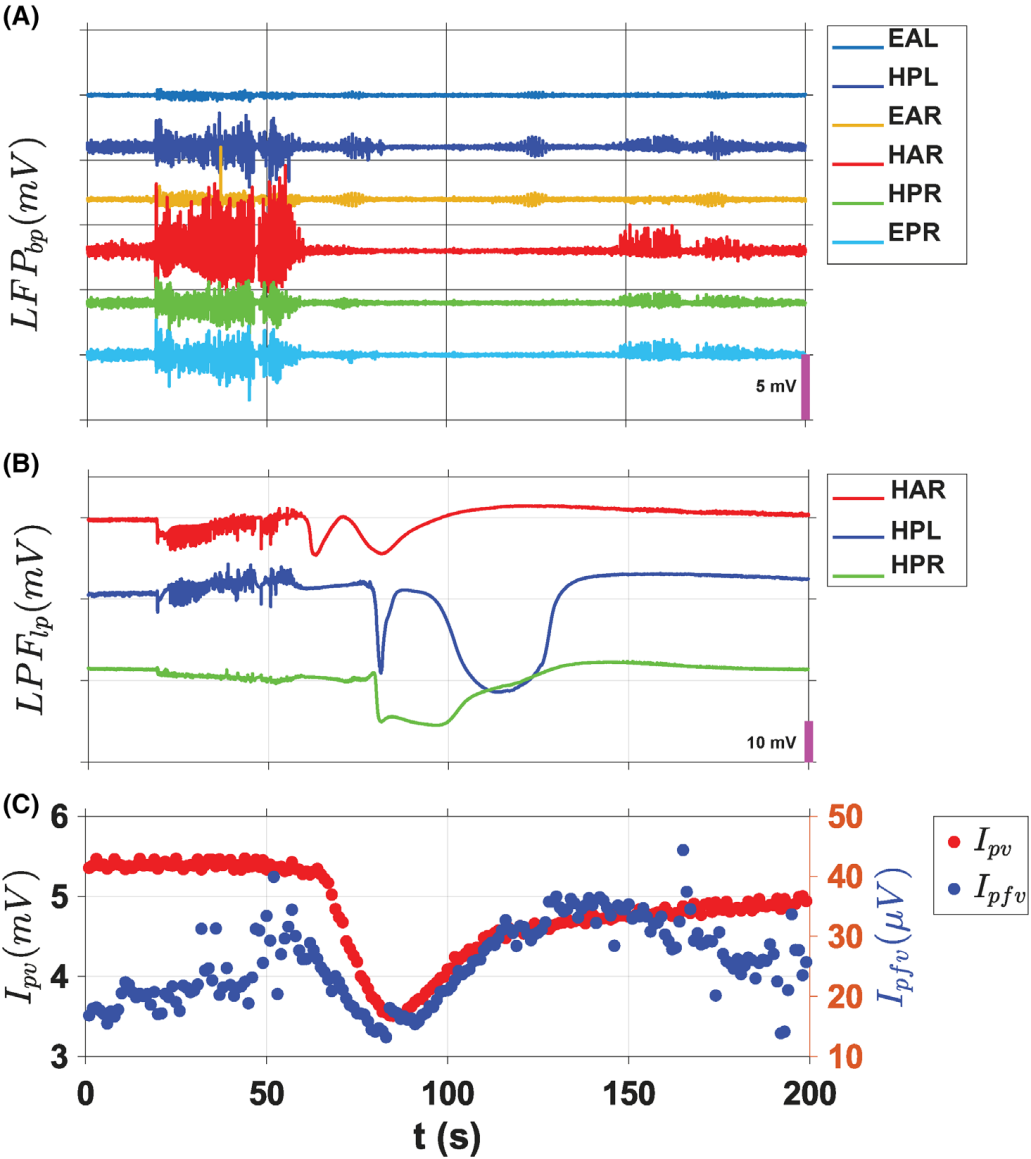
Representative recordings of hippocampal local field potential (LFP), electrocorticography (ECoG), and long pulse voltammetry (LPV) during a seizure to spreading depolarization transition. A 200‐s episode shows simultaneous recordings of LFP from hippocampal (HC) depth electrodes, ECoG from screws, and the oxygen‐sensing electrodes. (A) Band pass filtered ECoG and hippocampal LFP measurements. (B) Low pass filtered hippocampal LFP measurements. (C) LPV current response. *I*_pv_ (red dots, left y‐axis), LPV *I*_pfv_ (blue dots, right y‐axis). EAL, ECoG anterior left, anteroposterior (AP) −1.5, mediolateral (ML) −4 mm; EAR, ECoG anterior right, AP −1.5, ML 4 mm; EPR, ECoG posterior right, AP −6.5, ML 3 mm; HAR, HC anterior right, AP −2.5, ML 2.0, dorsoventral (DV) −3.2 mm); HPL, HC posterior left, AP −3.9, ML −2.2, DV −2.88 mm; HPR, HC posterior right, AP −3.9, ML 2.2, DV −2.54 mm; *I*_pfv_, LPV peak flat value; *I*_pv_, LPV peak value; t, time.

In a previous study, we demonstrated chronic oxygen sensing along with electrophysiological recordings via a representative 1‐h recording example.^38^ Here, we applied both CPA and LPV. Figure 3 shows a representative 200‐s recording episode from hippocampal (HC) depth electrodes, electrocorticographic (ECoG) screws, and LPV measures. Band‐pass filtered ECoG and HC local field potentials (LFPs) are shown in Figure 3A. Low pass filtered HC LFPs show SD initiation and propagation in the HC (Figure 3B). The SD propagation speed is 4.3 ± 2 mm/min (mean ± standard deviation, 44 events), consistent with previous studies.^6^, ^9^, ^51^, ^52^ The LPV measurement employed a 1‐Hz waveform with a 200‐ms pulse duration. *I*_pv_ (red dots, left y‐axis) and *I*_pfv_ (blue dots, right y‐axis) are shown in Figure 3C.

### 3.1 CPA current and LPV feature dynamics during normal SOV transitions

Using CPA in rats, previous studies have demonstrated that brain tissue oxygen levels increase during wakefulness and REM sleep and decline during NREM sleep.^53^, ^54^ The transition from NREM to wake is accompanied by a marked increase in oxygen, reflecting enhanced inspired oxygen and ventilation associated with wakefulness.^55^ Similarly, arousing stimuli elicit rapid elevations in brain oxygen, further supporting the sensitivity of CPA to state‐ and stimulus‐dependent changes in oxygen availability.^56^ Our previous result^38^ and current recordings via CPA show similar dynamics during transitions from NREM to REM and from NREM to wake with relatively smaller amplitudes but consistent and statistically significant changes across all rats. Compared with normal SOV CPA measures reported in Dash et al.,^53^ the smaller amplitude changes observed in our recordings likely reflect genuine physiological differences in the TeTX model. Additionally, the prior study was conducted in frontal and prefrontal cortical regions, whereas our measurements were recorded from the hippocampus. It is well established that changes in the vascular network and associated cell function lead to decreased oxygenation and impaired neurovascular coupling in the hippocampus compared to the cortex.^57^

**FIGURE 4:**
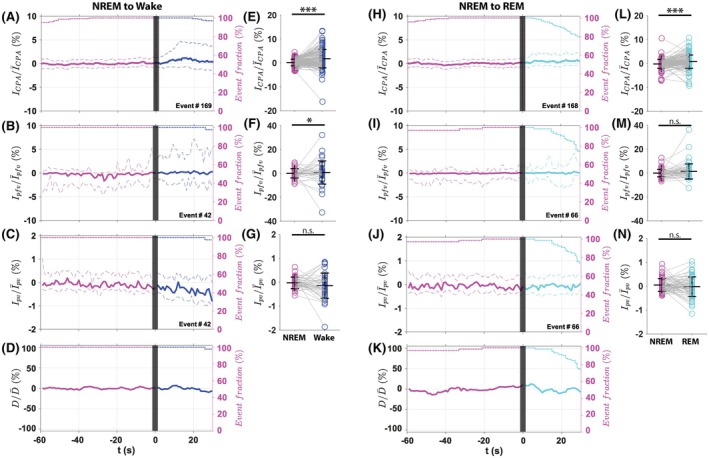
Constant potential amperometry current (*I*_CPA_) and long pulse voltammetry (LPV) feature dynamics during normal state of vigilance transitions. Normal state of vigilance transitions include non‐rapid eye movement (NREM) sleep to rapid eye movement (REM) sleep transition (NREM to REM), and NREM sleep to wakefulness transition (NREM to Wake). REM onset and wake onset are at time (t) = 0 (black bar). Each trace shows the median ± quantiles across observed transitions (left y‐axis, solid line ± dashed line, magenta: NREM, blue: Wake, cyan: REM). The event fraction (right y‐axis) shows the percentage of events at each time point and provides context for interpreting the ensemble‐averaged dynamics (right y‐axis; round‐dotted lines; magenta: NREM, blue: Wake, cyan: REM). During the NREM to wake transition, the *I*_CPA_ showed a significant increase (A, E). The LPV peak flat value (*I*_pfv_), comparable to CPA current (*I*_CPA_), showed a similar significant increase (B, F). The LPV peak value (*I*_pv_), reflecting local tissue oxygenation, decreased slightly (<1%) without significance (C, G). The effective diffusion coefficient (*D*_eff_) remained stable (D). During the NREM to REM transition, the *I*_CPA_ showed a significant increase (H, L). The *I*_pfv_ showed a similar but nonsignificant trend (I, M). LPV *I*_pv_ remained unchanged (J, N), and the *D*_eff_ remained stable (K). Statistical analyses confirmed significant CPA current changes (L), whereas LPV *I*_pfv_, although it showed a similar dynamic trend, did not show a significant change, possibly due to limited sample size (M). *p < 0.1, ***p < 0.001, and n.s., not significant.

Figure 4 shows the dynamics of CPA current and LPV features from NREM to wake and from NREM to REM. From NREM to wake, the *I*_CPA_ shows a significant increase (Figure 4A,E). The *I*_pfv_, comparable to the *I*_CPA_, shows a similar significant increase (Figure 4B,F). The *I*_pv_, reflecting local tissue oxygenation, decreases slightly without significance (Figure 4C,G). The *D*_eff_ remains stable (Figure 4D), indicating no ECS volume fraction (*α*) change.

From NREM to REM, the *I*_CPA_ increases significantly (Figure 4H,L). The *I*_pfv_ shows a similar but nonsignificant trend (Figure 4I,M), possibly due to limited sample size. The *I*_pv_ remains unchanged (Figure 4J,N), and the *D*_eff_ remains stable (Figure 4K), indicating no *α* change.

In summary, *I*_CPA_ and *I*_pfv_ showed significant increases in oxygen levels during normal SOV transitions. However, *I*_pv_ showed no significant change. The *D*_eff_ remained unchanged, indirectly indicating no significant alterations in *α* during normal SOV transitions.

### 3.2 CPA current and LPV feature dynamics during transitions from normal SOV to seizure and from seizure back to normal SOV

Previous optical and oxygen‐sensitive probe‐based studies report a transient “initial dip” in oxygen availability during seizures within the ictal focus, reflecting a rapid metabolic demand surge that briefly exceeds supply, whereas recordings from surrounding areas show increased oxygenation without this dip.^58^, ^59^, ^60^

**FIGURE 5:**
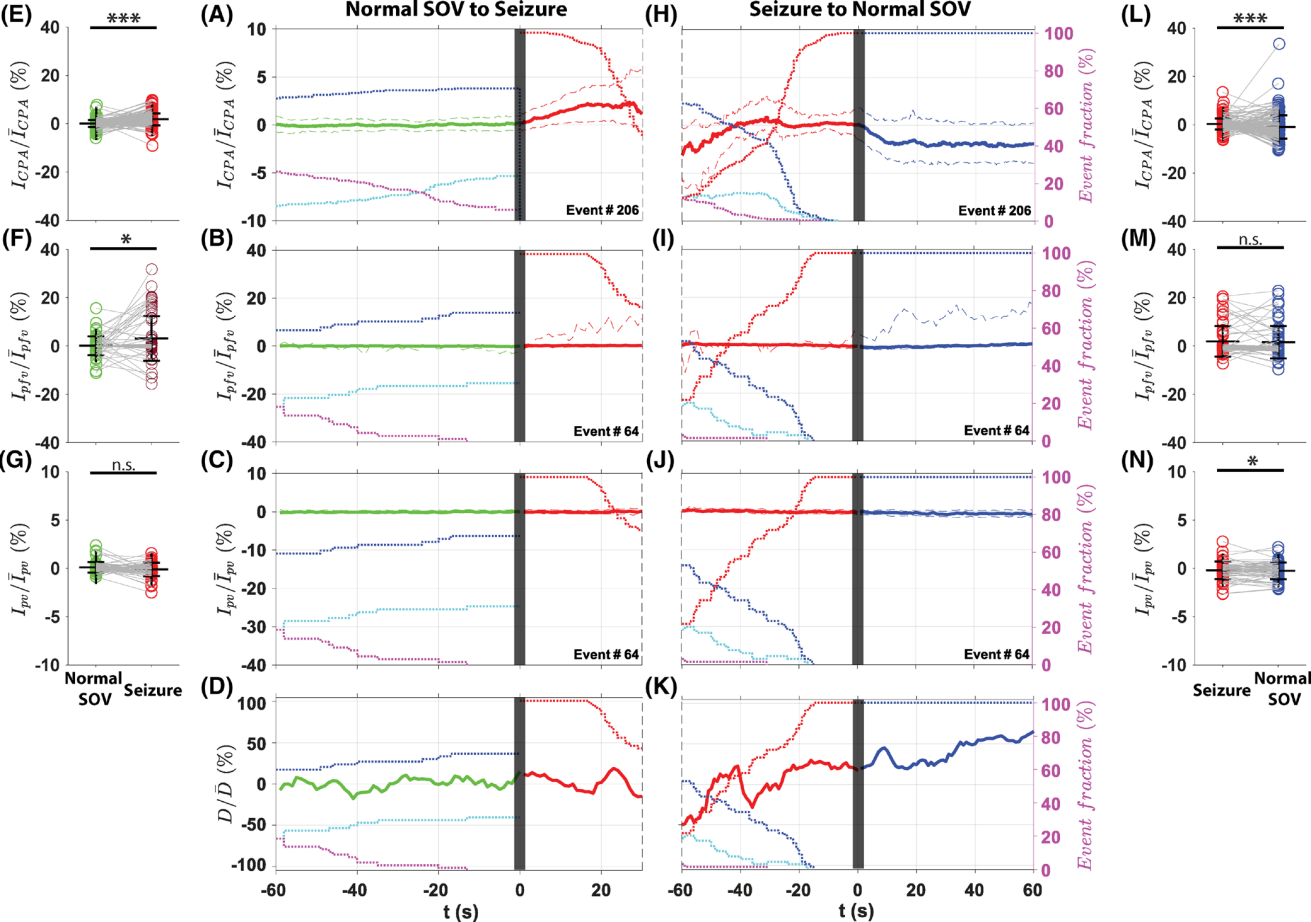
Constant potential amperometry current (*I*_CPA_) current and long pulse voltammetry (LPV) feature dynamics during transitions from normal state of vigilance (SOV) to seizure and from seizure back to normal SOV. Each trace shows the median ± quantiles across observed transitions (left y‐axis, solid line ± dashed line, green: normal SOV. red: seizure. blue: wake). The event fraction (right y‐axis) shows the percentage of events at each time point and provides context for interpreting the ensemble‐averaged dynamics (right y‐axis, dotted line, red: seizure, magenta: non‐rapid eye movement, blue: wake, cyan: rapid eye movement). Seizure onset and seizure offset are at time (t) = 0 (black bar). The *I*_CPA_ first increased significantly (A, E) and then decreased significantly after seizure offset during recovery (H, L). The LPV peak flat value (*I*_pfv_), which is comparable to the *I*_CPA_, showed a similar trend with a significant increase (B, F) followed by a nonsignificant decrease, likely due to limited sample size and higher noise levels (I, M). LPV peak value (*I*_pv_), reflecting local tissue oxygenation, remained unchanged during seizure (C, G) but showed a significant decrease after seizure offset (J, N). The effective diffusion coefficient (*D*_eff_) decreased during seizure and increased after seizure offset to wakefulness (D, K). *p < 0.1, ***p < 0.001, and n.s., not significant.

From normal SOV to seizure and back to normal SOV, the *I*_CPA_ increased significantly (Figure 5A,E) and then decreased significantly after seizure offset during recovery (Figure 5H,L). The *I*_pfv_, comparable to the *I*_CPA_, showed a similar significant increase (Figure 5B,F) followed by a nonsignificant decrease, likely due to higher noise levels (Figure 5I,M). The *I*_pv_, reflecting local tissue oxygenation, remained unchanged during seizure (Figure 5C,G) but showed a significant decrease after seizure offset (Figure 5J,N). The *D*_eff_ decreased during seizure and increased after seizure offset to wakefulness (Figure 5D,K). Notably, we did not find significant dynamic differences in *D*_eff_ across seizure onsets from different vigilance states (i.e., transitions from NREM, REM, or wakefulness to seizure). Accordingly, these transitions were analyzed as a single normal SOV to seizure condition, with the event fractions of NREM, REM, and wake shown on the right y‐axis.

The WE was implanted in the area close to but not at the TeTX injection site (i.e., ictal focus). We demonstrate an overall increased ictal oxygenation without the initial dip in either CPA or LPV recording, consistent with previous findings.^58^, ^59^, ^60^ Importantly, the *I*_pv_ showed no evidence of local oxygenation breakdown before seizure onset, indicating that peri‐ictal oxygenation followed the transition from normal SOV to seizure without an anticipatory deficit. We detected a postictal local oxygenation decrease, consistent with earlier studies reporting prolonged hypoxia.^61^ The *D*_eff_ dynamic change indirectly demonstrated an ictal ECS shrinkage aligning with prior studies; seizures in mice reduced *α* by ~35%,^62^ and a ~15% shrinkage has been reported during spontaneous epileptiform discharges in trauma‐injured rat neocortex.^63^

### 3.3 CPA current and LPV feature dynamics during transitions from normal SOV to seizure and to seizure‐associated SD

**FIGURE 6:**
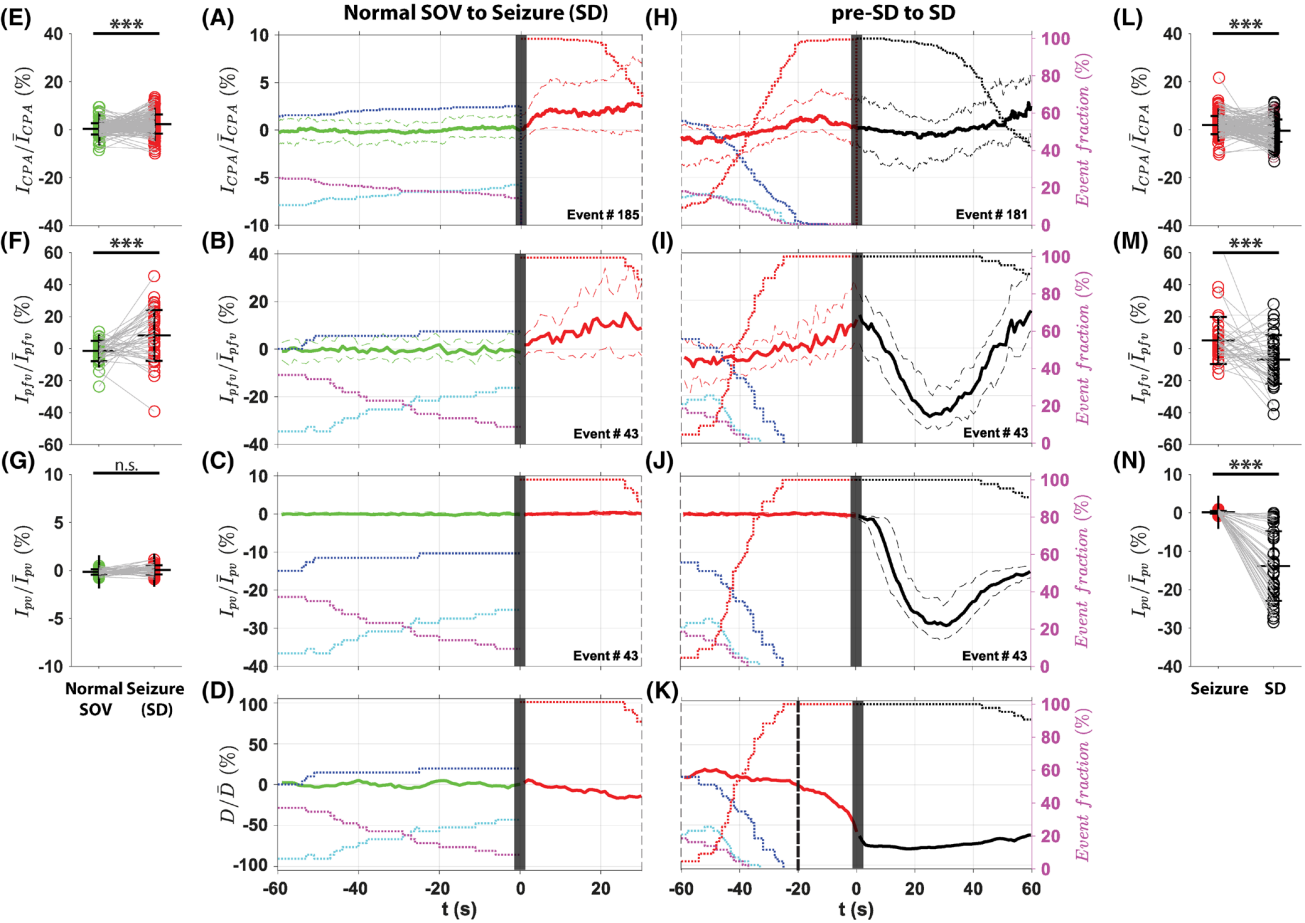
Constant potential amperometry current (*I*_CPA_) current and long pulse voltammetry (LPV) feature dynamics during transitions from normal state of vigilance (SOV) to seizure and to seizure‐associated spreading depolarization (SD). Each trace shows the median ± quantiles across observed transitions (solid line ± dashed line, green: normal SOV. red: seizure. black: SD). The event fraction (right y‐axis) shows the percentage of events at each time point and provides context for interpreting the ensemble‐averaged dynamics (right y‐axis, dotted line, black: SD, red: seizure, magenta: non‐rapid eye movement, blue: wake, cyan: rapid eye movement). Seizure onset and SD onset (seizure offset) are at time (t) = 0 (black bar). The *I*_CPA_ showed a significant increase during the transition from normal SOV to seizure (A, E) and a significant decrease during SD, recovering as the rate of SD event fraction decreased (H, L). The LPV peak flat value (*I*_pfv_) also showed similar dynamics across this transition. It first increased from normal SOV to seizure (B, F) and then decreased significantly during SD, returning to baseline as SD survival rates declined (I, M). However, the LPV peak value (*I*_pv_) did not show significant changes during the transition from normal SOV to seizure (C, G), but after the onset of SD, the *I*_pv_ decreased significantly (J, N). The effective diffusion coefficient (*D*_eff_) began to decline as the seizure event fraction increased before the SD onset, with the approximate onset of this decrease indicated by a vertical black dashed line at *t* = −20 s. During SD, the *D*_eff_ continued to drop by as much as 80% (D, K). ***p < 0.001, and n.s., not significant.

There are two pathways in our TeTX model following the seizure: return to a normal SOV or enter a SD. CPA current and LPV features from normal SOV to seizure, and to seizure‐associated SD, are shown in Figure 6.

The *I*_CPA_ increased significantly from normal SOV to seizure (Figure 6A,E), decreased significantly during SD, and recovered as the SD event fraction decreased (Figure 6H,L). The *I*_pfv_, comparable to the *I*_CPA_, showed similar dynamics; it first increased from normal SOV to seizure (Figure 6B,F), decreased significantly during SD, and returned to baseline as the SD event fraction declined (Figure 6I,M). However, the *I*_pv_ did not show significant changes from normal SOV to seizure (Figure 6C,G), but after SD onset, the *I*_pv_ decreased significantly during SD (Figure 6J,N), indicating that local oxygenation does not break down and behaves in a following role from normal SOV to seizure. Decreased local oxygenation during SD aligns with previous studies, in which direct tissue PO_2_ recordings revealed a rapid drop in local oxygen levels during SD, occasionally reaching near‐anoxic conditions, before partially recovering depending on vascular reactivity and the baseline perfusion state.^8^, ^19^

During the normal SOV to seizure transition, the *D*_eff_ began to decline as the seizure event fraction increased. Importantly, this decline occurred before the SD onset. After the SD onset, the *D*_eff_ continued to drop by as much as 80% during SD (Figure 6D,K). This result demonstrates that the ECS shrinkage happens before SD onset and may potentially play a leading role in the transition from seizure to SD. The result during SD is also consistent with a previous study describing a neuronal swelling during SDs.^8^

During the peri‐SD transition, *I*_CPA_ and *I*_pfv_ showed similar patterns, with a significant increase followed by a significant decrease. However, the *I*_CPA_ averages differed by only a few percent (Figure 6A,H), whereas the *I*_pfv_ showed larger changes of 15%–25% (Figure 6B,I). We demonstrated that *α* dropped by as much as 80% during SD (Figure 6K), indicating that *D*_eff_ cannot be assumed constant during this transition. Given that the *I*_pv_ has a much higher signal‐to‐noise ratio and is therefore the more reliable measure, the large *α* implies that *I*_CPA_ and *I*_pfv_ were corrupted by ECS volume shrinkage, which explains the difference between the two measurements.

### 3.4 Extracellular space dominates oxygen diffusion

The ECS volume fraction (*α*) change affects the *D*_eff_. From one state to another state (e.g., from seizure to SD), the *α* changes to *αʹ*. Cell centers remain unchanged, and the morphological change is shown with a larger *r* (Figure 1B). As with Equation (6), we get:

(7) $$D_{\mathrm{eff}}^{\prime }=\alpha^{\prime }D_{1}+\left(1-\alpha^{\prime }\right)D_{2}$$

where *αʹ* is the ECS volume fraction in the new state. Oxygen diffusion pathways distance from local to bulk, *d*, is assumed to be constant. We set *α* = .2 during normal SOV,^2^ and make an extreme assumption that *αʹ* = 0 during SD (Figure 6K). From Equations (6) and (7), we get *D*_2_ = 21 × *D*_1_, demonstrating that the ECS is the dominant oxygen diffusion pathway in the tissue and is altered dramatically by *α* change during SD.

## 4 DISCUSSION

Tissue oxygenation reflects the dynamic balance between oxygen delivery, utilization, tissue reactivity, and morphology under physiological conditions.^55^ CPA has been widely used for in vivo oxygen sensing. However, CPA‐based studies often assume a constant *D*_eff_ or cannot separate the *D*_eff_ effect from oxygen concentration changes.^43^ We introduce a 1D oxygen diffusion model showing that the *I*_CPA_ is a mixed signal of *C*_B_ and *D*_eff_. Using the LPV, we decouple these components, with the *I*_pfv_ comparable to the *I*_CPA_, and the *I*_pv_ reflecting local oxygen concentration. Using the *I*_pfv_ and the *I*_pv_, we derive *rv*, characterizing *D*_eff_ changes directly and *α* indirectly during different state transitions, including spontaneous seizure to seizure‐associated SD transition.

We demonstrated increased *I*_CPA_ and *I*_pfv_ recordings during transitions from NREM to wake, and from NREM to REM (Figure 4A,B,H,I), consistent with prior CPA works showing elevated oxygen during wakefulness and REM relative to NREM.^53^, ^55^ These increases reflect enhanced ventilation and oxygen intake upon arousal, together with local neurovascular adjustments that support increased neuronal activities.^56^, ^64^, ^65^ However, *I*_pv_ and *D*_eff_ indicated no significant change in local tissue oxygenation or *α* during normal SOV transitions (Figure 4C,D,J,K). This suggests that temporary increases in oxygen during behavioral arousal in normal SOV are significantly smaller or “buffered” by a baseline homeostatic mechanism with considerable metabolic demands, which helps maintain stable local oxygenation and ECS volume under normal physiological conditions.^64^, ^65^ The baseline buffering metabolism is also consistent with the tight coupling between local metabolism and cerebral blood flow, which stabilizes oxygen delivery across states.^66^, ^67^ Previous studies have indirectly demonstrated that although rapid stimulation‐induced oxygen dynamics can be detected with high temporal resolution methods such as CPA or fast oxygen electrodes, baseline oxygenation remains stable across physiological conditions.^54^, ^68^

Our findings during peri‐ictal and peri‐SD highlight dynamic changes in local oxygenation and ECS volume. During seizures, *I*_CPA_ and *I*_pfv_ increase significantly, consistent with previous studies (Figure 5A,B,H,I). However, LPV *pv* showed no significant breakdown of local oxygenation before seizure onset and during ictal period, indicating that peri‐ictal elevated metabolic demand was compensated by increased cerebral oxygen delivery through intact neurovascular coupling and local oxygenation was preserved despite the surge in neuronal activity.^58^, ^59^, ^60^ In parallel, we demonstrated decreased ECS volume during seizures (Figure 5D,K), consistent with prior studies of activity‐dependent ECS constriction. Such ECS shrinkage likely facilitates the accumulation of ECS potassium and neurotransmitters, amplifying excitability and promoting seizure propagation.^3^, ^69^, ^70^, ^71^, ^72^

From seizures to SDs, *I*_CPA_ and *I*_pfv_ decreased significantly (Figure 6A,B,H,I). However, *I*_pv_, reflecting local oxygenation, remained stable before SD onset (Figure 6C) and dropped significantly during SD (Figure 6J). This finding demonstrates the following. First, local oxygenation does not break down during seizures before SD onset, suggesting that impaired oxygen supply is not the primary trigger for SD initiation. Second, the collapse of local oxygenation during SD reflects a near‐anoxic state, consistent with direct tissue PO_2_ recordings that show dramatic oxygen depletion during SD^8^, ^19^ and with the elevated metabolic demand required for recovery.^73^, ^74^ This severe oxygen decline also reflects a failure of neurovascular coupling during SD.^75^ It was demonstrated indirectly in stroke patients using intraoperative hybrid photoacoustic and ultrasonic imaging to monitor blood oxygenation during SD.^76^ A previous rat study showed that SD was accompanied by an initial hypoperfusion (~75% of baseline), followed by transient hyperemia (~220% of baseline) and subsequent oligemia.^28^ Researchers reported a large drop in tissue PO_2_ after SD onset with a less pronounced decrease in CBF, followed by significant increases in both tissue PO_2_ and CBF.^30^, ^31^ In a mouse study, researchers showed that worsening mismatches between oxygen supply and consumption drive the initiation and propagation of SD.^77^ The high metabolic demand during SD arises from massive, near‐complete depolarization of neurons and glia, collapse of ionic gradients, and recruitment of ATP‐dependent pumps to restore homeostasis.^4^, ^78^ When combined with impaired or spatially restricted vascular responses, this overwhelming demand–supply mismatch results in sustained hypoxia.

Researchers described ECS shrinkage during SD.^8^ The ECS volume has been reported to decrease from 20% to approximately 5% because of the water influx due to ion changes, leading to intracellular hyperosmolality.^5^, ^24^, ^79^, ^80^ We demonstrated that the ECS volume decrease began during seizure, before the SD onset, and continued to decrease dramatically up to 80% during SD (Figure 6D,K), suggesting that significant ECS shrinkage (or cell swelling) may play a leading role in inducing SD during seizures. The restricted ECS diffusion may exacerbate metabolic stress by further limiting oxygen and substrate availability and triggering SD.^62^, ^63^

Together, these results underscore the fundamentally different local oxygenation and ECS volume dynamics of seizures versus SD. Seizures, in intact tissue, appear metabolically sustainable; neurovascular coupling remains preserved, and compensatory hyperemia supports oxygen delivery despite concurrent ECS constriction. In contrast, SD imposes an overwhelming metabolic burden associated with near‐complete neuronal and glial depolarization, collapse of ionic gradients, and dramatic ECS shrinkage. This burden overwhelms vascular compensation, leading to profound hypoxia. Our results may help explain why seizures are often reversible, whereas seizure‐associated SDs are strongly linked to neuronal injury and adverse clinical outcomes.^16^, ^81^

Our results also demonstrate that the ECS is the dominant pathway for oxygen diffusion in brain tissue, consistent with prior studies. Classic models of tissue oxygenation first described oxygen diffusion from capillaries into the surrounding extracellular fluid as the primary route for substrate delivery.^40^ Following studies have shown that ECS volume fraction and tortuosity critically shape the efficiency of oxygen transport.^3^ Experimental work further supports this view, with measurements of cortical oxygen dynamics indicating that ECS diffusion governs both the spatial extent and temporal resolution of oxygen delivery to active neuronal populations.^82^ Although oxygen can also cross cell membranes and diffuse through the cytoplasm to reach mitochondria,^83^, ^84^ this intracellular pathway plays a secondary role compared to the ECS pathway. Our results, showing that ECS shrinkage during seizures and seizure‐associated SD strongly constrains oxygen availability, reinforce the view that ECS diffusion is the principal determinant of local oxygen dynamics. Recent work has suggested an additional and rapid membrane‐associated diffusion pathway for oxygen.^85^ However, from a three‐dimensional tissue perspective, the total flux of freely diffusing oxygen molecules through this pathway is substantially smaller than through the ECS and is expected to contribute minimally to the bulk diffusion signal measured by CPA or LPV. Moreover, this membrane‐associated pathway is unlikely to change substantially across normal SOV, seizures, or SD, and should not confound the relative, state‐dependent changes reported here.

We addressed a fundamental gap in understanding seizure‐associated SD mechanisms by applying CPA and LPV for long‐term, continuous recordings in freely behaving animals with intact autoregulation, providing implications for SUDEP and other epilepsy‐related comorbidities.

## AUTHOR CONTRIBUTIONS

Jiayang Liu and Bruce J. Gluckman designed and constructed the hardware and designed the research. Jiayang Liu analyzed the data and wrote the paper.

## FUNDING INFORMATION

This work is partially supported under NIH awards R01EB019804 and R01EB014641.

## CONFLICT OF INTEREST STATEMENT

The authors report no conflict of interest. We confirm that we have read the Journal's position on issues involved in ethical publication and affirm that this report is consistent with those guidelines.

## Supporting information


Figure S1.

Figure S2.

Data S1.

## Data Availability

The data that support the findings of this study are available on request from the corresponding author.
