# Supplementary figures and images for: Tissue oxygenation dynamics during transition from seizure to spreading depolarization in rat brain
Source: Epilepsia. 2026 Mar 20;67(6):3171–84. doi: 10.1002/epi.70207 (PMC13285254; doi:10.1002/epi.70207)

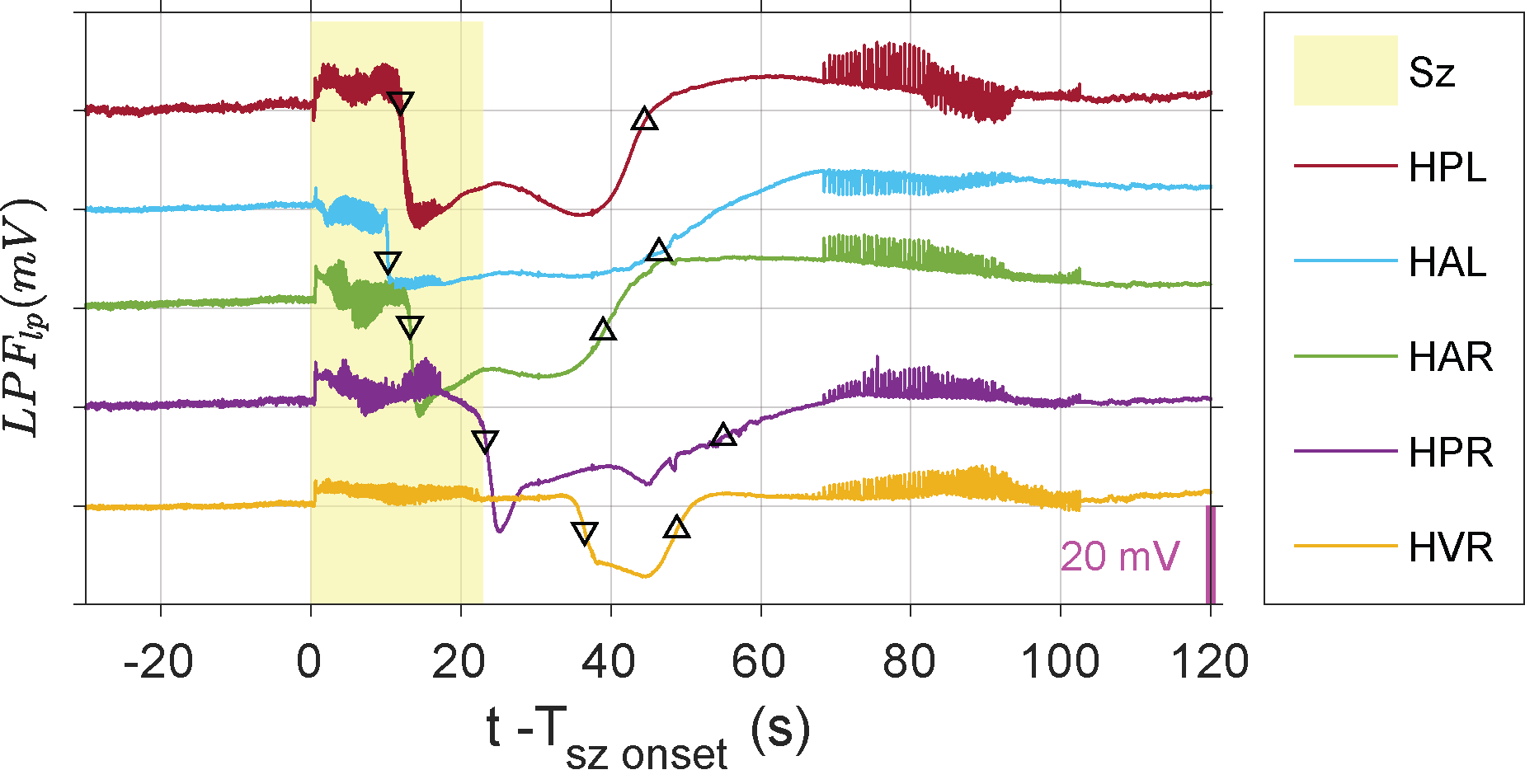

Supplement: Supplementary file 1 — Figure S1. [file EPI-67-3171-s002.tif]

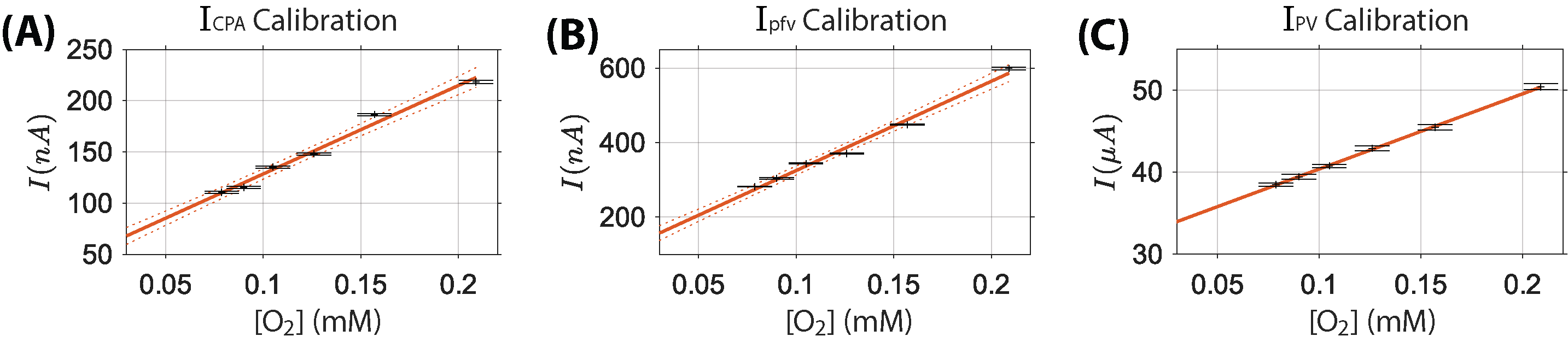

Supplement: Supplementary file 2 — Figure S2. [file EPI-67-3171-s003.tif]
