# Supplementary material for: Tissue oxygenation dynamics during transition from seizure to spreading depolarization in rat brain
Source: Epilepsia. 2026 Mar 20;67(6):3171–84. doi: 10.1002/epi.70207 (PMC13285254; doi:10.1002/epi.70207)
Supplement: Supplementary file 3 — Data S1. [file EPI-67-3171-s001.docx]

**Supplementary Materials for:**

Tissue Oxygenation Dynamics During Seizure to Spreading Depolarization in Rat Brain

**Authors:**

Jiayang Liu^1,2*^, Bruce J. Gluckman^1,2,3,4^

**Affiliations:**

1 Center for Neural Engineering, Pennsylvania State University; University Park, PA 16802, USA.

2 Department of Engineering Science and Mechanics, Pennsylvania State University; University Park, PA 16802, USA.

3 Department of Neurosurgery, Pennsylvania State College of Medicine; Hershey, PA 16802, USA.

4 Department of Biomedical Engineering, Pennsylvania State University; University Park, PA 16802, USA.

* Corresponding authors email addresses:

Jiayang Liu, ljylxl@gmail.com

**Electrode implantation**

For hippocampus local field potential (LFP) recordings, we employed 50 µm diameter micro-reaction chamber (µRC) electrode pairs (1) with tips 125 ∼ 250 µm apart dorsally. Stainless steel screws were employed for measuring ECoG. For electrochemical recordings using the CPA or LPV, we employed a 200 μm diameter platinum (Pt) wire with PET (polyethylene terephthalate) plastic film coating (A-M Systems, Inc.) as the working electrode (WE), a custom-made Ag/AgCl electrode as the reference electrode (RE), and a screw electrode as the counter electrode (CE). The Ag/AgCl electrode was created by inserting a 127 µm diameter silver wire into a 155 µm diameter polyamide tube (A-M Systems, Inc.) filled with Ag/AgCl ink (CI-4001 Silver/Silver Chloride/Vinyl, Nagase America LLC).

All surgeries were conducted under deep anesthesia using Ketamine (90 mg/kg) and Xylazine (15 mg/kg). A heating pad was utilized under the animal to maintain a constant body temperature of 37°C. Rats were secured in a stereotaxic frame with ear bars and given pre-operative Buprenorphine Ethiqa XR (3.25 mg/kg) for pain relief. Lidocaine (<5 mg/kg) was injected subcutaneously at the incision site. Burr holes were drilled based on stereotaxic coordinates relative to bregma (Mouse Atlas, Paxinos and Franklin, 2001 (Paxinos 2001)). Specific targets and naming conventions were organized into three categories: (**1**) Hippocampal LFP recordings via µRC electrode pairs at HAR (hippocampal anterior right, AP -2.5, ML 2.0, DV -3.2 mm), HPL (hippocampal posterior left, AP -3.9, ML -2.2, DV -2.88 mm), and HPR (hippocampal posterior right, AP -3.9, ML 2.2, DV -2.54 mm). (**2**) ECoG recordings via stainless steel screw electrodes at EFL (ECoG frontal left, AP 2, ML -3 mm), EFR (ECoG frontal right, AP 2, ML 3 mm), EAL (ECoG anterior left, AP -1.5, ML -4 mm), EAR (ECoG anterior right, AP -1.5, ML 4 mm), EPL (ECoG posterior left, AP -6.5, ML -3 mm), and EPR (ECoG posterior right, AP -6.5, ML 3 mm). (**3**) Oxygen sensing via O₂-WE (oxygen-sensing working electrode, AP -2.5, ML -2, DV -3.1 mm) in the hippocampus, and O₂-Ref (oxygen-sensing reference electrode, AP -5.15, ML -5.35 mm) at the cortex. The locations of the working electrode (WE) and reference electrode (RE) were selected based on pilot experiments using this TeTX model with multiple depth electrodes implanted in each hippocampus (2, 3). From these experiments, we found that SD events consistently appeared to propagate from a region close to the spatial offset between the O₂ measurement site and the toxin injection site. This region was close to the seizure focus and identified as the most frequent site of SD initiation. Previous studies have also demonstrated this spatial dissociation between the seizure focus and the site of SD initiation (4). One representative example is shown in **Figure S1** (2), where HAL (hippocampal anterior left) is the target of WE implantation.

Rats were prepared under the TeTX model (5), which has been used to induce seizures in mice and rats (6). Researchers have characterized the mechanisms of action of the toxin as well as seizure development and progression (5, 7-10). Usually, after the toxin injection, the animal will begin to experience spontaneous seizures within 10 days. The procedures for toxin injection and electrode implantation are outlined in (11). Briefly, 10 to 13 nano-grams of tetanus toxin (Santa Cruz Biotechnology, CAS 676570-37-9) dissolved in 1.3 microliters phosphate-buffered saline (PBS) mixed with 2% bovine serum albumin (BSA) were injected into the left ventral hippocampus (AP -5.15, ML -5.35, DV -6.1 mm) through a 30-gauge flexible cannula over 15 minutes with additional 30 minutes for tissue relaxation. The custom-made Ag/AgCl RE was placed in the cortex along the same trajectory, minimizing further damage to the cortex. LFP recording depth electrodes, ECoG screw electrodes (with EFL and EFR being the ground and reference electrode), WE, and RE were secured in place on the skull and electrically isolated via dental cement. After surgery, rats were returned to individual standard autoclave-ready cages with free access to food and water and maintained at a 12-hour light-dark cycle with lights on between 7 am and 7 pm. We allowed a seven-day post-surgery recovery before initiating recordings.

**Oxygen-sensing electrode calibration**

Noble metal electrodes have been widely used for oxygen sensing and remain a well-established standard for in vivo and chronic recordings, including commercially available products (e.g., Pinnacle Technology). We used a 200-μm-diameter Pt wire with PET insulation (A-M Systems, Inc.) as the working electrode (WE). Although carbon-based electrodes are also common in chronic oxygen sensing and can offer reduced sensitivity to certain redox interferents, custom-built carbon electrodes often exhibit greater variability in effective surface area and contact geometry, leading to inconsistent calibration. In contrast, Pt electrodes provide a more reproducible and well-characterized electrochemical surface, which was important for reliable cross-animal comparisons in our experiments. Potential fouling of the bare Pt surface was addressed through *in vitro* calibration before implantation.

The *in vitro* oxygen-sensing calibration setup for the WE is described in previous work (2). We implemented a three-electrode electrochemical cell with a Pt wire WE, an Ag/AgCl pellet RE, and a Pt plate CE. Oxygen levels were controlled by mixing air-saturated PBS with Nitrogen (N_2_)-saturated PBS, and electrodes were submerged in the air-saturated solution first, followed by incremental addition of the N₂-saturated solution. Experiments were conducted at room temperature with the PBS solution at pH 7.48 (pH Meter, Model P771. Anaheim Scientific). CPA and LPV waveforms were applied by the electrochemical instrument described in (2). The resulting oxygen concentrations were derived from standard stoichiometric calculations.

The CPA calibration result is shown in **Figure S2A**. In brain tissue, oxygen concentration, partial pressure of oxygen (*PO_2_*), and oxygen tension are mutually related and can be derived from one another. Oxygen concentration is expressed in moles per liter (mol/L), measuring dissolved oxygen. *PO_2_* reflects the amount of free oxygen molecules and equals the pressure that oxygen would exert if it occupied the space by itself (12). The oxygen concentration (Millimole/L, mM) was used in this study. The LPV *pfv*, comparable to the CPA measurement as discussed in the modeling section, exhibits a similar calibration result (**Figure S2B**). Given that the calibration process occurs in a PBS solution, we assume that the effective oxygen diffusion coefficient remains constant. This assumption has been supported by the *pv* calibration result shown in **Figure S2C**.

Amperometric oxygen measurements can be influenced by local changes in pH and redox state, both of which are known to vary during SD events (13). Previous *in vivo* studies of SD have shown that extracellular pH undergoes a characteristic biphasic change, with an initial alkalinization followed by pronounced acidification (14-19). To assess whether such pH fluctuations could confound our oxygen measurements, we performed *in vitro* calibration experiments in which pH was systematically varied across the full range reported *in vivo* during SD (20). These results demonstrated that even large pH changes did not produce significant alterations in the CPA signal, indicating that the oxygen measurements reported here are not substantially affected by physiologically relevant extracellular pH dynamics.

**Data analysis and statistics**

Recorded data include LFP (*µ*RC electrodes), ECoG (screw electrodes), three-axis head-acceleration signal, and tissue oxygenation signals (CPA and LPV). Data were processed offline using custom-written MATLAB (MathWorks Inc.) programs for re-referencing, filtering, spectral analysis, and behavior annotation. LFPs were band-pass filtered at 0.5 ∼ 125Hz to highlight field potential and seizure dynamics. Seizure and SD detection methods followed (2). **Seizures were detected** by a stereotypical increase in spectral power that initiates with a sentinel spike followed by a burst of 9 ~ 16Hz hippocampal spikes (21), spreads through the cortex, and ends with a sharp decrease in spectral power. Seizures shorter than 10 seconds or spaced apart less than 10 minutes were excluded. **SDs were determined** from at least one depth electrode (HAR, HPL, or HPR) after a 0.5Hz low pass filter. SD onset was defined by a downward crossing of a 7.5mV threshold relative to the 3s value before. SD offset was detected by an upward crossing of a threshold defined as 2mV above the SD onset potential. **SD propagation speed** was determined by dividing the physical distance between electrodes HAR and HPR by the difference in time indices corresponding to their SD onset crossings. Only animals with ‘clean’ signals on HAR and HPR were used. Normal SOV was clarified as: REM sleep characterized by a spectral peak in the theta (4 ∼ 7 Hz) frequency band of hippocampal LFP and by an absence of acceleration except during brief muscle twitches; NREM sleep characterized by maximal delta band (0.5 ∼ 4 Hz) power and by an absence of acceleration; wake characterized by the accelerometer activity (22, 23). Transitions include NREM to REM, NREM to wake, normal SOV to seizure to normal SOV, and normal SOV to seizure to SD.

CPA currents were preprocessed by removing large artifacts or filling 'nan' for small artifacts. After a 10Hz low-pass filter, the current time course was baseline-normalized and down-sampled from 1kHz to 20Hz. Baseline normalization was performed by subtracting and dividing by the 60s pre-state mean, manifesting the dynamic change and setting the trace start at 0%. The median normalized current with quantile was plotted from a 60s pre-state to a post-state, with a display cutoff at 60s for SD events, and 30s for other post-states.

LPV current responses are “discrete” pulses. After applying the same preprocessing steps, the *pv* and the *pfv* were extracted from each pulse. The *pv* was measured at the current maximum during the bias potential transition from *E_1_* to *E_2_*. The *pfv* was calculated as the average current recorded 10–20 ms before the next potential transition. Using *pv* and *pfv*, we extracted *rv*, characterizing the change of *D_eff_*. LPV *pv, pfv,* and *rv* from all transitions were baseline z-score normalized to get a time course from a 60s pre-state to post-state transition, with a display cutoff at 60s for SD events, and 30s for other post-states.

To assess whether the distributions of values differed between defined temporal epochs spanning state transitions, we applied the two-sample Kolmogorov–Smirnov (K-S) test (Matlab function kstest2). For each transition, data were segmented into a 5-s pre-state window and a 5-s post-state window, offset from the transition onset to avoid contamination by the transition itself. Specifically, the pre-state window spanned −10 to −5 s relative to transition onset (defined as time 0), and the post-state window spanned +5 to +10 s. The distributions of values within these two windows were compared using the two-sample K–S test. The mean ± standard deviation values of the pre- and post-state windows were reported for visualization and summary purposes with significance levels set at p < 0.001 or p < 0.05.

**REFERENCES**

1. Shanmugasundaram B, Gluckman BJ. Micro-reaction chamber electrodes for neural stimulation and recording. Annu Int Conf IEEE Eng Med Biol Soc. 2011;2011:656-9.

2. Liu J, Gluckman BJ. A DC-sensitive video/electrophysiology monitoring unit for long-term continuous study of seizures and seizure-associated spreading depolarization in a rat model. bioRxiv. 2025:2025.02.04.635811.

3. Fatemeh Bahari PS, Jiayang Liu, John Kimbugwe, Carlos Curay, Steven J. Schiff, Bruce J. Gluckman. Seizure-associated spreading depression is a major feature of ictal events in two animal models of chronic epilepsy. 2020.

4. Mitlasóczki B, Gómez AG, Kamali M, Babushkina N, Baues M, Kück L, et al. Hippocampal spreading depolarization as a driver of postictal ambulation. Science Translational Medicine. 2025;17(816).

5. Jefferys JGR, Walker MC. Tetanus Toxin Model of Focal Epilepsy. Models of Seizures and Epilepsy. 2006:407-14.

6. Mellanby J, George G, Robinson A, Thompson P. Epileptiform syndrome in rats produced by injecting tetanus toxin into the hippocampus. J Neurol Neurosurg Psychiatry. 1977;40(4):404-14.

7. Jefferys JG, Borck C, Mellanby J. Chronic focal epilepsy induced by intracerebral tetanus toxin. Ital J Neurol Sci. 1995;16(1-2):27-32.

8. Sunderam S, Chernyy N, Mason J, Peixoto N, Weinstein SL, Schiff SJ, Gluckman BJ. Seizure modulation with applied electric fields in chronically implanted animals. Conf Proc IEEE Eng Med Biol Soc. 2006;2006:1612-5.

9. Sunderam S, Chernyy N, Peixoto N, Mason JP, Weinstein SL, Schiff SJ, Gluckman BJ. Seizure entrainment with polarizing low-frequency electric fields in a chronic animal epilepsy model. J Neural Eng. 2009;6(4):046009.

10. Sedigh-Sarvestani M, Thuku GI, Sunderam S, Parkar A, Weinstein SL, Schiff SJ, Gluckman BJ. Rapid eye movement sleep and hippocampal theta oscillations precede seizure onset in the tetanus toxin model of temporal lobe epilepsy. J Neurosci. 2014;34(4):1105-14.

11. Sedigh-Sarvestani M, Thuku GI, Sunderam S, Parkar A, Weinstein SL, Schiff SJ, Gluckman BJ. Rapid Eye Movement Sleep and Hippocampal Theta Oscillations Precede Seizure Onset in the Tetanus Toxin Model of Temporal Lobe Epilepsy. Journal of Neuroscience. 2014;34(4):1105-14.

12. Subczynski WK, Swartz HM. EPR Oximetry in Biological and Model Samples. In: Eaton SR, Eaton GR, Berliner LJ, editors. Biomedical EPR, Part A: Free Radicals, Metals, Medicine, and Physiology. Boston, MA: Springer US; 2005. p. 229-82.

13. Farkas E, Rose CR. A dangerous liaison: Spreading depolarization and tissue acidification in cerebral ischemia. J Cereb Blood Flow Metab. 2025;45(2):201-18.

14. Somjen GG. Mechanisms of spreading depression and hypoxic spreading depression-like depolarization. Physiol Rev. 2001;81(3):1065-96.

15. Kraig RP, Cooper AJ. Bicarbonate and ammonia changes in brain during spreading depression. Can J Physiol Pharmacol. 1987;65(5):1099-104.

16. Kraig RP, Ferreira-Filho CR, Nicholson C. Alkaline and acid transients in cerebellar microenvironment. J Neurophysiol. 1983;49(3):831-50.

17. Somjen GG. Acidification of Interstitial Fluid in Hippocampal-Formation Caused by Seizures and by Spreading Depression. Brain Research. 1984;311(1):186-8.

18. Tong CK, Chesler M. Endogenous pH shifts facilitate spreading depression by effect on NMDA receptors. Journal of Neurophysiology. 1999;81(4):1988-91.

19. Xiong ZQ, Stringer JL. Extracellular pH responses in CA1 and the dentate gyrus during electrical stimulation, seizure discharges, and spreading depression. Journal of Neurophysiology. 2000;83(6):3519-24.

20. Liu J, Gluckman B. Tissue Oxygenation Dynamics During Seizure to Spreading Depression Transition in Rat Brain: The Pennsylvania State University; 2021.

21. Finnerty GT, Jefferys JGR. 9-16 Hz oscillation precedes secondary generalization of seizures in the rat tetanus toxin model of epilepsy. Journal of Neurophysiology. 2000;83(4):2217-26.

22. Sunderam S, Chernyy N, Peixoto N, Mason JP, Weinstein SL, Schiff SJ, Gluckman BJ. Improved sleep-wake and behavior discrimination using MEMS accelerometers. J Neurosci Meth. 2007;163(2):373-83.

23. Bahari F, Ssentongo P, Schiff SJ, Gluckman BJ. A Brain-Heart Biomarker for Epileptogenesis. Journal of Neuroscience. 2018;38(39):8473-83.

**FIGURE LEGENDS**

**Figure S1: Example of seizure-associated spreading depolarization event propagation in the hippocampus.** A recording episode showing a transition from the normal state of vigilance (SOV) to seizure, followed by a seizure-associated spreading depolarization (SD) event. Low pass filtered hippocampal LFP measurements (HPL, HAL, HAR, HPR, and HVR) show SD initialization and propagation. SD shows a two-directional propagation after initialization: on the left, SD propagated from HAL posteriorly to HPL, and on the right, SD crossed to HAR, then to HPR, and finally to HVR. HAL (hippocampal anterior left), HAR (hippocampal anterior right), HPL (hippocampal posterior left), HPR (hippocampal posterior right), HVR (hippocampal ventral right). Modified from (2) with permission.

**Figure S2: Oxygen-sensing working electrode calibration via CPA and LPV.** Linear fitting results of current response versus oxygen concentration (X-axis, oxygen concentration; Y-axis, current). (**A**) CPA calibration result. (**B**) LPV peak flat value (*pfv*) calibration result. (**C**) LPV peak value (*pv*) calibration result. Each data point was derived from the addition of N2-saturated PBS solution. For each addition, we recorded data for approximately 5 minutes, and each value was calculated from the middle 80 seconds of this recording to eliminate any transients associated with the addition process.
